# Commentary: Do health care workforce, population, and service provision significantly contribute to the total health expenditure? An econometric analysis of Serbia

**DOI:** 10.3389/fphar.2017.00033

**Published:** 2017-02-06

**Authors:** Mihajlo Jakovljevic, Mirjana Varjacic

**Affiliations:** ^1^Health Economics and Pharmacoeconomics, The Faculty of Medical Sciences, University of KragujevacKragujevac, Serbia; ^2^Gynaecology Department, The Faculty of Medical Sciences, University of KragujevacKragujevac, Serbia

**Keywords:** health expenditures, serbia, forecasts, cost drivers, health care workforce, population aging

With interest we have been reading the recent article entitled: “Do health care workforce, population, and service provision significantly contribute to the total health expenditure? An econometric analysis of Serbia” (Santric-Milicevic et al., 2016). Authors provided a decent piece of econometric modeling analysis. Here they evaluated on a supply side a considerable burden imposed by the pool of professional health workforce employees. Authors have pointed out that “*the growth of the health workforce number in the previous year has strongly contributed to the growth of total health expenditure in Serbia from 2003 to 2011.”* Time series modeling approach adds to the scarce body of evidence on health expenditure determinants in post-socialist Eastern European societies.

However, there are few significant gaps in the article deserving to build them up with the omitted evidence. In the explanatory background section a lot has been said about the structural reforms of the national health system. These reforms were to a large extent led by the external consultancies provided by the World Bank through the Health Project Serbia^1^ and Second Serbia Health Project^2^ whose recommendations were adopted by frequently changing national governments since the early 2000s. These changes took place for well over a decade and a half. Some of them, like the attempt to establish the national health technology assessment (HTA) agency, did that with a very limited success (Jakovljevic et al., 2011). Understanding of these background processes is necessary to catch a glimpse of the big picture in the Balkan health systems (Bredenkamp et al., 2011). Actual lack of confidence of health care professionals in many of these reform initiatives has been shown in large scale national surveys of clinical physicians (Jakovljevic et al., 2016a). Others point out to the significant growth of inequalities in terms of medical care access and affordability among the ordinary citizens in recent years (Radevic et al., 2016).

In the section entitled “Drivers of health expenditure in the Republic of Serbia” little has been said about the microeconomic drivers of local medical spending. Mostly top-down national health reports were cited in this chapter. However, there is a strong body of quantified, bottom-up assessments on real-world costs of care in Serbian health system. Over the past 15 years academic health economists have identified core drivers of high costs of inpatient and outpatient medical care. Innovative pharmaceuticals imaging diagnostics, interventional radiology procedures, dental care and radiotherapy in oncology are among the highest impact ones (Jakovljevic et al., 2013, 2014, 2015a; Rankovic et al., 2013; Rančić et al., 2015). A set of regional cost of illness analysis have been conducted as well pointing out that non-communicable prosperity diseases sharing the highest budget impact were high risk pregnancies, diabetes, depression, alcohol addiction, HIV/AIDS, COPD and cancer (Jakovljevic et al., 2008; Biorac et al., 2009; Jovanovic and Jakovljevic, 2011; Dagovic et al., 2014; Cupurdija et al., 2015; Jakovljević et al., 2015; Arnlöv, 2016). Good example as well is the structural trend analysis on long term health expenditure evolution in Serbia published few years ago (Jakovljevic, 2014). Since the focus of the source article is health spending we should emphasize the evolving role of evidence based resource allocation in all of former Yugoslavia's republics (Jakovljevic, 2013). When explaining the growing availability of professional staff in Serbia, authors properly notice: “*Throughout that period, the accessibility of physicians, nurses, and midwives per 10 000 population has increased by 14 % but with significant inequity across districts.”* However, they miss to mention the scale of geographical inequality in density of staff distribution. Most of this pool of physicians, nursing and associated medical staff is concentrated in four largest cities with heavily neglected rural periphery. Heavy migration of skilled labor force toward rich urban cores happens due to higher living standards and stronger employment prospects (Stilwell et al., 2004). Some relief could be found in a skillful financing and provision of primary care services throughout the country (Konstantinović et al., 2012). This exceptional centralization of health workforce capacities in capital cities is not unique to Serbia. It presents a landmark of many Eastern European health systems (Simai, 2006). This is even more prominent in dental workforce and pharmaceutical spending evolution going in different directions in Eastern and Western Europe alongside former Cold War borderlines (Jakovljevic et al., 2016b,c).

In conclusion, authors noticed the role of the growing size of an aging population coupled with strong emigration net rates (Santric-Milicevic et al., 2014). Probably the most typical challenge of rising portion of elderly to the long term sustainability of health care financing could be found in the eldest of nations—Japanese one (Ogura and Jakovljevic, 2014). Nevertheless we should not forget the broad perspective of the population aging in Europe which might be bringing some opportunities together with the difficulties (Jakovljevic, 2015a). Keeping in mind Eastern European perspective, there has been straight forward evidence on connection between the health expenditure long term dynamics and the extension of human longevity (Jakovljevic et al., 2015b). Other major drivers of spending such as the chronic illnesses could be efficiently tackled by more effective provision and financing of hospital care (Mihailovic et al., 2016). Even more promising is the investment into the preventive screenings and other primary outpatient care and life style interventions as was the case in nearby Hungary (Sándor et al., 2016). One of the cost-effective strategies is improved quality of pharmacotherapy nationwide in terms of better patient compliance (Gustafsson et al., 2011). Another complimentary approach is dissemination of good clinical practice guidelines controlling the rate of drug adverse events in hospital and outpatient care (Godman et al., 2013). Pharmaceuticals acquisition costs could be contained by ongoing transformation of the local market targeted to strengthen generic substitution of brand name drugs (Woerkom et al., 2012; Howard et al., 2015). Authors are right in their rather pessimistic opinion on prospects of national health expenditure growth in Serbia up to 2020. But to have these long term projections more reliable, due to their peculiarity, we should look toward more similar health systems such as those of the leading BRICS emerging markets (Jakovljevic, 2015b). Many of these nations share the historical legacy of health care establishments similar to that of Serbia to a large degree. Getzen's excess growth model in forecasting health expenditures gives us hints of what is going on in these nations up to 2025 (Jakovljevic et al., 2016d). Since they have been burdened with similar issues and constraints far earlier and too a far larger extent, small post-socialist nations might be capable to learn valuable lessons for their own future (Jakovljevic et al., 2016e).

## Author contributions

MJ and MV have jointly designed the research question, prepared the manuscript and revised it for important intellectual content. They share equal authorship responsibility.

## Funding

We acknowledge the Ministry of Education Science and Technological Development of the Republic of Serbia for Grant ON 175 014 supporting the underlying studies laying grounds for this Commentary article. Publication was not contingent upon Ministry's approval or cenzorship.

### Conflict of interest statement

The authors declare that the research was conducted in the absence of any commercial or financial relationships that could be construed as a potential conflict of interest.

## Footnotes

^1^Health Project (Serbia). http://www.worldbank.org/projects/P077675/health-project-serbia?lang=en. Accessed: 01 October 2016.

^2^Second Serbia Health Project. http://www.worldbank.org/projects/P129539/second-serbia-health-project?lang=en. Accessed: 01 October 2016.
